# Ovarian Inguinal Hernia in Premenopausal Women: A Case Report

**DOI:** 10.7759/cureus.20846

**Published:** 2021-12-31

**Authors:** Subhra Samantroy, Amaresh Mishra, Jyochnamayi Panda, Pramila Jena

**Affiliations:** 1 Obstetrics and Gynecology, Kalinga Institute of Medical Sciences, Bhubaneswar, IND; 2 Surgery, Kalinga Institute of Medical Sciences, Bhubaneswar, IND

**Keywords:** ultrasonography, oophorectomy, premenopausal women, ovary, inguinal harnia

## Abstract

A 34-year-old woman presented to the hospital emergency department complaining of abdominal pain for four days, more so in the left iliac fossa, and six episodes of vomiting for one day. Physical and sonographic examinations revealed an inguinal hernia containing a twisted gangrenous ovary with fallopian tube and partially developed uterus. The patient underwent an emergency hernia exploration with left oophorectomy, repositioning of the uterus with a fallopian tube, and herniorrhaphy without complications. A preoperative diagnosis based on history, physical examination, and ultrasonography allows for accurate surgical planning and corrective surgery without complications.

## Introduction

Inguinal hernias tend to be more common in males (90%) than females (10%), and usually occur with omentum or small bowel herniation [1]. Most Inguinal hernias have either a congenital or acquired form, but adult male Inguinal hernias are mostly acquired. Over the course of a lifetime, almost 25% of men develop this condition and less than 2% of women. The inguinal hernia containing ovary and fallopian tube is more common in children and is rare among women of reproductive age, as they are mostly caused by congenital abnormalities of the female genital tract [2].

Uterus, ovary, and fallopian tubes are rarely present with an inguinal hernia. A retrograde analysis of 1950 cases of operable inguinal hernia revealed that the vermiform appendix was present in 0.51% of the cases, ovaries and fallopian tubes in 2.9%, and urinary bladder in 0.36% of the cases [3].

## Case presentation

A 34-year-old woman from a low-socioeconomic background, partially literate, presented at Pradyumna Bal Memorial Hospital, Kalinga Institute of Medical Sciences (KIMS), Bhubaneswar, with pain in abdomen, mostly in the left iliac fossa, and six episodes of vomiting for one day. The patient was fine four days ago, but started experiencing pain in the abdomen that was colicky in nature, more so in the left iliac fossa and radiating to her inguinal area, which she treated at a local hospital for three days and then referred to our hospital as the pain still persisted. She developed vomiting which was non-projectile and bilious for one day. Her menstrual history was suggestive of primary amenorrhea and she was married for 16 years. The past medical history was suggestive of hypothyroid for which she was taking thyroid hormones (levothyroxine) for the last two years, and she did not have any relevant family history. Her bowel and bladder habits were normal. On examination, she was conscious, oriented, and hemodynamically stable except for tachycardia with a pulse of 96/minute. In abdominal examination, there was generalized tenderness, guarding, and rigidity. Bowel sound was diminished. Redness, local rise of temperature, and tenderness were there in the left inguinal area. In a few hours, progressive swelling of 3×2 cm appeared in the left inguinal area, which was uniform, soft in consistency, warm, tender, non-pulsatile, non-reducible, and cough reflex was absent. Other abdominal examinations were normal. On inspection, there was a dimpling in the vaginal area but secondary sexual characteristics were normal. Other systemic examinations were normal. Her hemoglobin, hematocrit, and total platelet count were within normal limits, whereas the total leucocyte count (TLC) was raised (15870/cm), with differential count of neutrophil 78%, lymphocyte 17%, eosinophil 4%, basophil 1%, and other routine blood tests like renal function test, liver function test, blood sugar, viral markers like HIV, hepatitis B surface antigen A, hepatitis C antigen, and ECG came out to be normal. Emergency ultrasonography (USG) had revealed a twisted left ovary herniating along the canal of Nuck. The right ovary was normal, seen in the ovarian fossa, and uterus could not be visualized.

**Figure 1:**
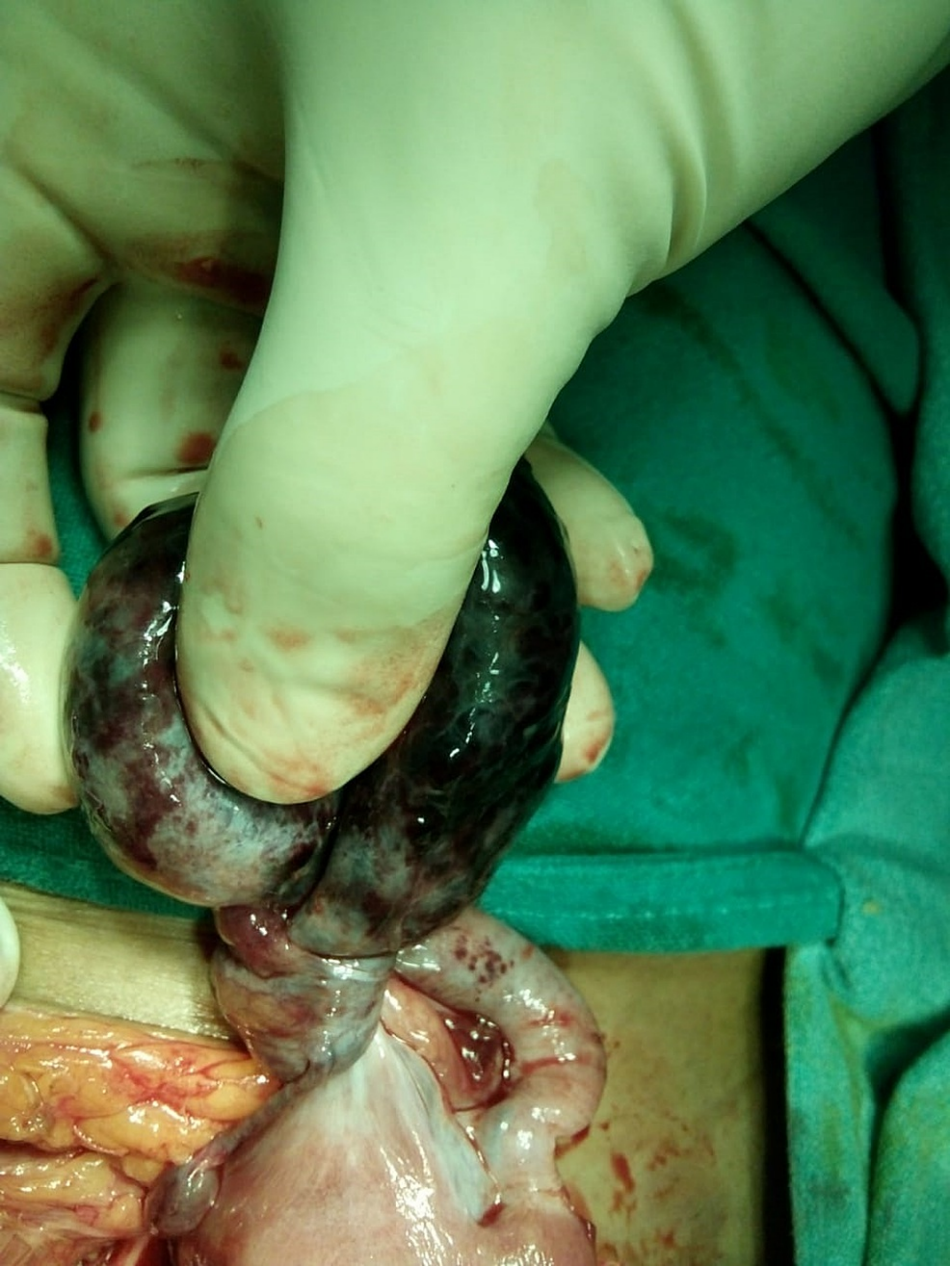
Twisted ovarian inguinal hernia.

**Figure 2:**
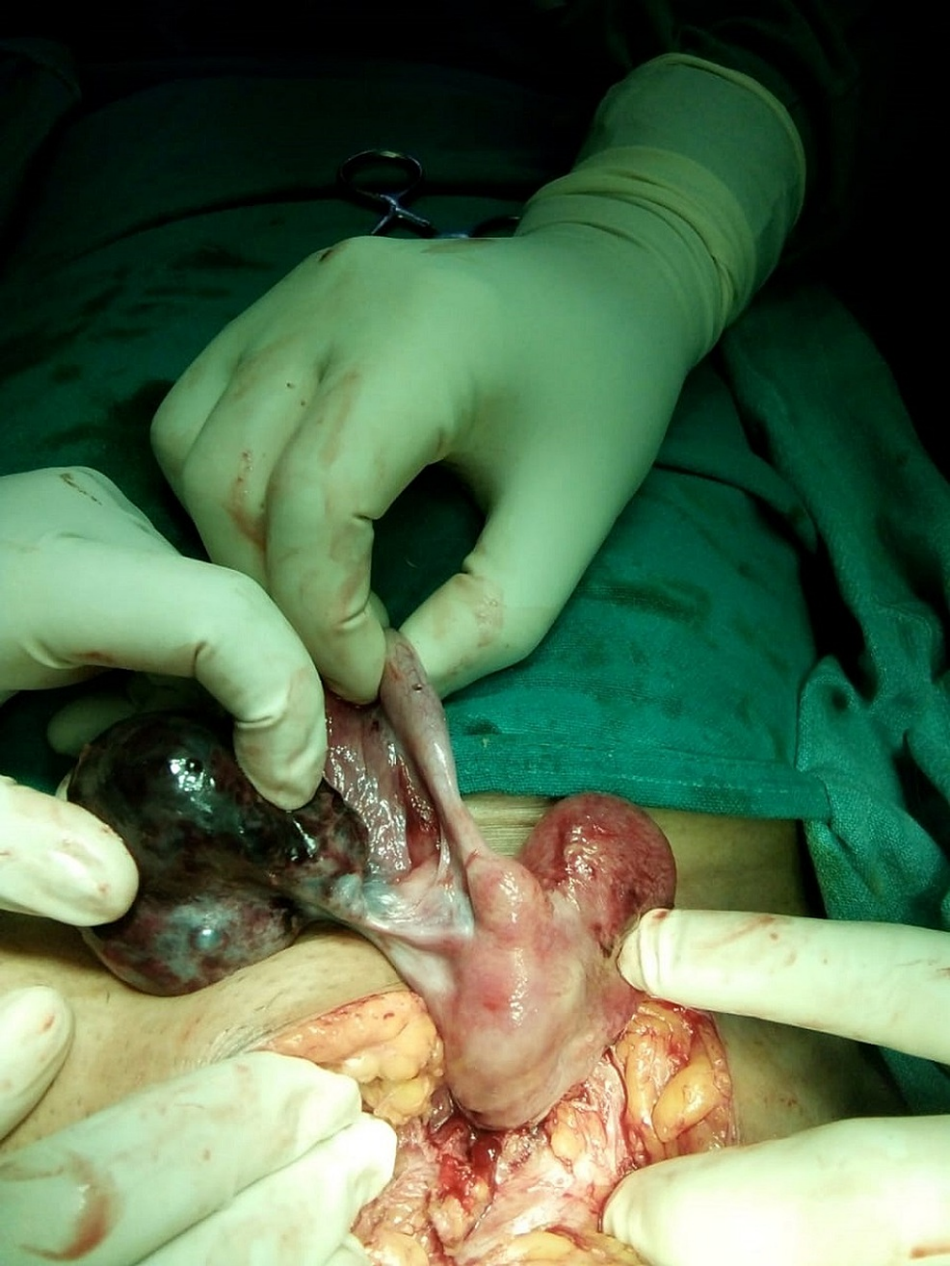
Untwisted ovarian inguinal hernia.

A case of strangulated inguinal hernia was diagnosed, and the emergency hernia exploration was done where the hernia sac was found to contain left side twisted gangrenous ovary with a partially developed uterus and bilateral fallopian tubes (Figure 1). After untwisting twisted hernia content left side oophorectomy was done and the fallopian tube with uterus was reposed to the abdominal cavity (Figure 2). A herniorrhaphy was done with Prolene suture #1 (Cornelia, GA: Ethicon Inc.) and closure of wound was done with a subcuticular drain. The drain was removed on the third postoperative day and the rest of the postoperative period was uneventful. The histopathological report came out with benign serous cystadenoma of the ovary with hemorrhagic infarct.

## Discussion

As per the literature, ovary and fallopian tube as content of inguinal hernia is rare in women of reproductive age group [2]. In contrast, most cases of gonadal hernias were reported in the pediatric age group in association with other genital tract anomalies. The reported incidence of its occurrence is 71% in children under five years and 30% in adolescents or women in the reproductive age group [3]. Thomson offered the hypothesis that when there is a failure of fusion of the Mullerian ducts, leads to excessive mobility of the ovaries, increases the chance of herniation of the entire uterus, ovary, and fallopian tube into the inguinal canal [4]. On the other hand, Fowler theorized that elongated ovarian suspensory ligaments were the primary cause of ovarian inguinal hernia [5]. Okada et al. also suggested that the weakness in the broad ligaments or ovarian suspensory ligaments can contribute to ovarian herniation into the inguinal ring [6]. There have been case reports of different unusual contents in inguinal hernia sacs, including parts of the genitourinary tract. Yao et al. suggested that in premenopausal women the morphological characteristics of the ovary in the hernia sac can be assessed through sonographic examinations, which provide information on the ovarian function. Sonographic imaging of the affected area combined with intra-abdominal or transvaginal ultrasonography revealing the absence of one of the two ovaries from the pelvis should enhance the diagnosis of an inguinal hernia [7]. The above ultrasonographic characteristics are similar to our case. Although ovaries are not commonly encountered by surgeons, a high index of suspicion is required to avoid delaying in diagnosis and treatment. It was reported that about 4-37% of female inguinal hernias have been found intra-operative present with non-reducible ovaries. Ovarian torsion and infarction have been encountered in 2-33% of these patients as found in our case which necessitates treating all cases even when asymptomatic [8]. Twisted ovarian cysts can be dealt effectively with the help of laparoscopy with concomitant repair of inguinal hernia if the diagnosis is made preoperatively [9]. This was not possible in our patient as our preoperative diagnosis was a strangulated inguinal hernia with suspicion of twisted ovary as its content. Differential diagnoses of the inguinal region swelling in the female are varied which may include direct inguinal hernia, soft tissue tumors (sarcoma, leiomyoma, or lipoma), abscess collection, enlarged lymph nodes, or hydrocele [10]. The diagnosis can be confirmed easily by ultrasonography scan in most cases. Treatment is done by reduction of the contents followed by ligation at the high level of the sac then closure of deep inguinal ring and finally re-supporting of the posterior wall of the inguinal canal by a non-absorbable mesh. This could not be done in our case as it was strangulated inguinal hernia containing gangrenous twisted ovary for which we did oophorectomy with reposition of the uterus and fallopian tubes followed by high ligation of sac and herniorrhaphy with non-absorbable polypropylene suture without mesh repair.

As the patient was from low socioeconomic background and from a remote place, we could not follow up with the patient postoperatively for further evaluation which is the major limitation of this report.

## Conclusions

Although this study is considered to be a very rare entity, the possibility of ovarian hernia should be kept in mind in a female patient presenting with an irreducible swelling in the inguinal region to avoid serious complications. Ultrasonography will confirm the content in most of the cases. Whenever suspected, it must be treated as a surgical emergency.
